# Asciminib antagonizes transplantable BCR::ABL1-positive lymphoid blast crisis in vivo by targeting malignant stem cells

**DOI:** 10.1038/s41375-024-02320-9

**Published:** 2024-06-21

**Authors:** Nicolas Chatain, Julian Baumeister, Marcelo A. Szymanski de Toledo, Dickson W. L. Wong, Siddharth Gupta, Kristina Pannen, Bärbel Junge, Tim H. Brümmendorf, Peter Boor, Steffen Koschmieder

**Affiliations:** 1 Department of Hematology, Oncology, Hemostaseology, and Stem Cell Transplantation, Faculty of Medicine, RWTH Aachen University, Aachen, Germany; 2 Center for Integrated Oncology Aachen Bonn Cologne Düsseldorf (CIO ABCD), Aachen, Germany; 3 Institute of Pathology, RWTH Aachen University Clinic, Aachen, Germany

**Keywords:** Preclinical research, Oncogenes, Targeted therapies

## Abstract

Philadelphia chromosome-positive (Ph+) lymphoid blast crisis (BC), emanating from chronic myeloid leukemia (CML), is a fatal disease with limited treatment options. Asciminib (ABL001) is a novel selective allosteric inhibitor of the ABL kinase with high efficacy against TKI-resistant BCR::ABL1. In this study, we demonstrate significant suppression of an aggressive B-lymphoblastic disease and restoration of normal hematopoiesis in an inducible transgenic mouse model of p210-BCR::ABL1-positive CML-BC. Molecularly, asciminib treatment significantly reduced *BCR::ABL1* transcripts to background levels, demonstrating its ability to suppress BCR::ABL1-induced disease. Furthermore, asciminib treatment normalized the long-term repopulating hematopoietic stem cell (LT-HSC) population in the BM, suggesting the selective targeting of malignant LT-HSCs. This was supported by secondary transplantation experiments, resulting in absence of BC in a proportion of mice. Importantly, none of the secondary transplanted mice that received further asciminib treatment developed leukemia. Sanger sequencing of the *BCR::ABL1* myristoyl pocket region of both treatment-naïve and treated mice demonstrated a high mutational load. However, there was no indication of asciminib-specific mutations. These promising findings highlight the potential of asciminib as a drug that targets BC stem cells and as an alternative stand-alone or combinatorial therapy for first-line treatment of CML BC or Ph+ acute lymphoblastic leukemia.

## Introduction

The oncogene BCR::ABL1 is a key player in chronic myeloid leukemia (CML), as well as in acute lymphoblastic leukemia (ALL), collectively known as Philadelphia chromosome-positive (Ph+) neoplasms. ABL-directed tyrosine kinase inhibitors (TKIs) such as imatinib and second-generation TKIs have proven to be effective against BCR::ABL1, achieving deep long-term remission in most chronic phase (CP) CML patients [1]. However, responses in blast crisis (BC) and Ph+-ALL are much less pronounced, requiring combination therapies or allogeneic hematopoietic stem cell transplantation [2]. In addition, TKI-resistant BCR::ABL1 mutations pose challenges, especially in monotherapy [3]. Importantly, in Ph+-ALL, there is considerable evidence for the presence of pre-existing ABL gene mutation at diagnosis and before the onset of TKI therapy in 7–37% of patients [4–7].

Asciminib (ABL001), a novel allosteric BCR::ABL1 inhibitor, targets the myristoyl binding pocket, with promising response in CML-CP third-line treatment after ≥2 previous TKIs [8]. However, data on asciminib’s efficacy in Ph+ BC and ALL are scarce, with reports indicating the emergence of resistance mutations [9].

This study utilized a tetracycline (tet)-inducible p210BCR::ABL1 transgenic mouse model [10] to assess asciminib’s effectiveness in BCR::ABL1-induced lymphoid BC (lyBC), a rapid and fatal disease. Secondary transplants and successive asciminib treatments were employed to analyze the malignant stem cell population and the emergence of drug-resistant mutations. The results indicate asciminib’s efficacy in alleviating the malignant B-cell phenotype and *BCR::ABL1* burden. Additionally, our findings confirm that the *BCR::ABL1* kinase domain (KD) and myristoyl binding pocket exhibit genetic instability in BC and are prone to base substitutions.

## Methods

For a full description of all reagents and methods, see Supplemental Methods.

### Mouse experiments

All experiments involving mice were approved by the local authorities of North Rhine-Westphalia, Germany. The SCLtTA-BCR::ABL1 double transgenic (dtg) mouse model has been described previously [10]. The procedure of primary and secondary transplantations is described in Fig. S1A, B, and Supplemental Methods.

### Flow cytometry and used antibodies

Peripheral blood (PB), bone marrow (BM), and spleen cells were stained with respective antibodies in PBS containing 2% fetal calf serum (FCS) at 4 °C for 30 min. Used antibodies are listed in Table S1. Flow cytometry measurements were conducted with a FACS Gallios system (Beckman Coulter, Brea, CA) and analyzed with FlowJo Software (version 10.8).

### Sequencing of the *BCR::ABL1* KD in single clones

As the FVB/N SCLtTA-BCR::ABL1 mouse strain carries multiple copies of human *BCR::ABL1* cDNA in its genome, PCR was performed to amplify the respective DNA section, the fragments were cloned into TOPO vectors using the TOPO™ TA Cloning™ Kit (Thermo Scientific, Waltham, MA), and Sanger sequencing was performed.

### RNA isolation and RT-qPCR

RNA was isolated from unfractionated BM and splenocytes according to manufacturer’s protocol (Macherey-Nagel, Düren, Germany). RT-qPCR was performed using the 7500 Fast Real-Time PCR System (Applied Biosystems by Life Technologies, Paisley, UK) with the SYBR Selected Master Mix for CFX (Applied Biosystems). *BCR::ABL1* expression was calculated as mean percentage of *Gapdh*. Used primers are listed in Table S2.

### Data analysis

Graphical representation and statistical analyses were performed with Prism 10 (GraphPad, CA, USA). Applied statistical tests are given in the figure legends. *p* values < 0.05 were considered statistically significant (*p < 0.05, **p < 0.01, ***p < 0.001, ****p < 0.0001).

## Results and discussion

### Rapid normalization of lymphoid blast crisis and increased survival upon asciminib treatment

**Fig. 1:**
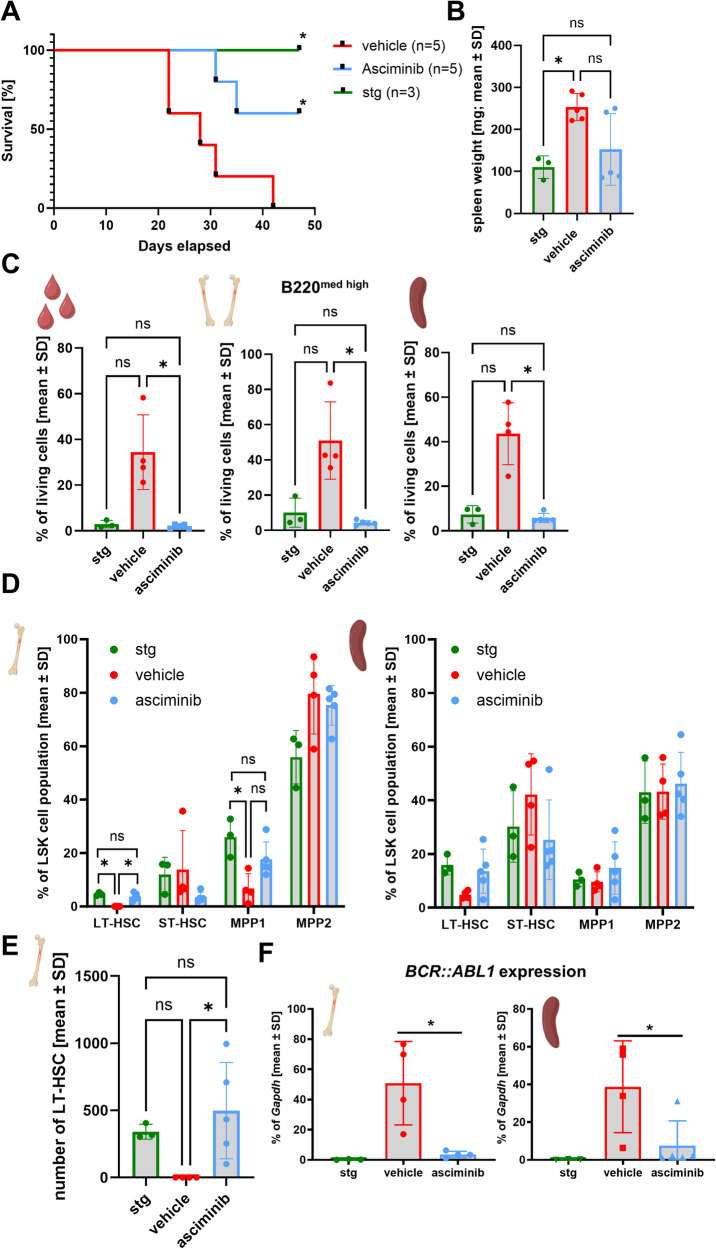
Lymphoid blast crisis is blocked by ABL001 treatment. **A** Log-rank (Mantel-Cox) test demonstrates significantly (*p* = 0.0259) higher survival of ABL001-treated mice or single-transgenic (stg) mice (*p* = 0.0123) in comparison to vehicle controls (*n* = 5 vehicle and ABL001; *n* = 3 stg), 49 days after transplantation. **B** Spleen weight of vehicle- and ABL001-treated, as well as single-transgenic (stg; *n* = 3) mice, were compared using a One-way ANOVA test (Kruskal–Wallace and Dunn’s multiple comparisons test). **C** Percentage of B220 medium-high (med-high) cells in peripheral blood, bone marrow, and spleen was analyzed by flow cytometry. One-way ANOVA test (Kruskal–Wallace and Dunn’s multiple comparisons test) was used for statistical analysis. **D** In the lin- Sca1+ cKIT+ (LSK) cell population, percentage of the LT-HSCs, ST-HSCs, MPP1, and MPP2 cells was analyzed in bone marrow and spleen. One-way ANOVA test (Kruskal–Wallace and Dunn’s multiple comparisons test) was used for statistical analysis. **E** Absolute cell number of LT-HSC in BM. One-way ANOVA test (Kruskal–Wallace and Dunn’s multiple comparisons test) was used for statistical analysis. **F** *BCR::ABL1* expression, normalized to *Gapdh*, in the bone marrow and spleen of transplanted mice. Mann–Whitney-*U*-test was used for evaluating significance using ABL001 as reference. *p < 0.05, **p < 0.01, ns not significant.

One week after removal of tet in the transplant recipient mice, asciminib or vehicle treatment was started for 5 weeks. After the first week of therapy, the phenotype was evaluated in the PB. While dtg mice in the vehicle-treated cohort developed lyBC, characterized by a medium-high B220/CD45.1 cell population in PB, asciminib-treated mice did not show any signs of lyBC development (Fig. S2A, B). At the same time, CD11b+/Gr1+ granulocytes and CD45.1+/CD3+ T-cells, which were suppressed by the lyBC cells in the vehicle-treated group, were increased in the asciminib-treated mice (Fig. S2B). After this initial assessment, mice were then followed for additional 4 weeks, and survival of asciminib-treated mice was significantly (*p* = 0.0067) increased in comparison to vehicle-treated mice (Fig. 1A). Spleen weight of the diseased vehicle group was significantly increased (*p* = 0.0193) in comparison to single-transgenic (stg) control mice, and asciminib treatment reduced spleen weight in three out of five mice (Fig. 1B). Meanwhile, asciminib reduced the aberrant B cell population in BM (*p* = 0.013), spleen (*p* = 0.0213) and PB (*p* = 0.0216) in all mice (Fig. 1C). In the BM of treated mice, the decrease of lyBC cells was associated with a significant increase of CD11b+/Gr1+ granulocytes to control levels (Fig. S2C). In the spleen, an increase of CD11b+/Gr1+ cells was observed, which may explain the heavier spleen in two of five mice, which also showed the highest percentage of CD11b+/Gr1+ cells. HE staining confirmed disturbed spleen architecture in vehicle-treated mice, which was reverted by asciminib treatment (Fig. S3A). A significant expansion of megakaryocytes in the spleen of BCR::ABL1-expressing mice was partly normalized by asciminib (Fig. S3A, B). Staining for B220 in BM and spleen also revealed restoration of more terminally differentiated B cells upon drug administration (Fig. S3A). These findings demonstrate the potent efficacy of asciminib in lyBC and are in line with the first clinical data of a combination study (NCT03595917) of asciminib with dasatinib and/or prednisone [9].

### Asciminib has disease-modifying potential by selectively targeting the mutated clone

Recent analyses postulate that B-ALL is not maintained by immature leukemia stem cells (LSC) but by leukemic lymphoid progenitors with aberrant self-renewal capability [11, 12]. Therefore, the stem cell compartment was analyzed to evaluate the effect of asciminib on most immature hematopoietic cells (Fig. S4A).

While the percentage of unselected lineage-negative (lin-) Sca-1+/cKIT+ (LSK) cells was increased by asciminib treatment in the BM (Fig. S4B, left), their total cell count was unaltered (Fig. S4B, right). The number of LSK cells was increased (*p* = 0.0194) in the spleen of vehicle-treated but not asciminib-treated mice (*p* = 0.2049) (Fig. S4C). Importantly, asciminib treatment blocked exhaustion of long-term hematopoietic stem cells (LT-HSC), which was observed in lyBC mice, restoring the relative and absolute numbers of LT-HSC to control levels in BM (*p* = 0.0405 and *p* = 0.024, respectively) and spleen (not significant) (Fig. 1D, E). In contrast, numbers of short-term HSC (ST-HSC), MPP1, and MPP2 were unaltered in the BM (Fig. S4D). The ablation of the lyBC phenotype was associated with a significant reduction of *BCR::ABL1* transcripts in BM and spleen (Fig. 1F), and the spleen weight correlated significantly with *BCR::ABL1* in the BM (Fig. S4E).

In summary, asciminib demonstrated high efficacy against the BCR::ABL1-induced lyBC phenotype, normalizing the LT-HSC compartment, and reducing oncogene expression, reflecting strong suppression of the malignant clone. Of note, multipotent lymphoid-committed progenitors and common lymphoid progenitors were not analyzed, and we can therefore not comment on the effects of asciminib on these progenitors in this study.

### Asciminib retained potent anti-leukemic activity upon secondary transplantation

To investigate whether asciminib treatment hampers the repopulating capacity of BCR::ABL1-positive stem cells, spleen cells of treated and untreated mice were transplanted into secondary recipients. Spleen cells were used for secondary transplantation on the basis of our previous findings that pre-induced spleen cells exhibit improved disease transplantability in comparison to BCR::ABL1-positive BM cells [13]. Subgroups of the secondary recipients were further treated with asciminib, aiming to increase mutational pressure (Fig. S1B).

**Fig. 2:**
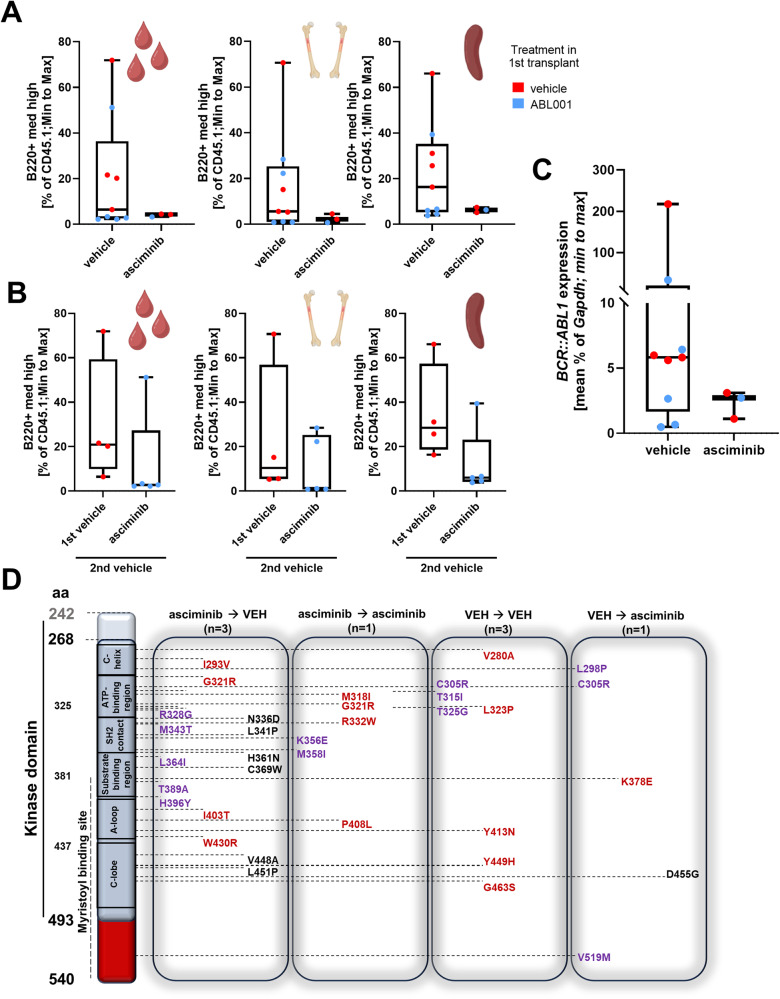
Asciminib retains potent antileukemic activity in secondary transplants. **A** Percentage of B220 medium-high (med-high) cells in peripheral blood, bone marrow, and spleen of the secondary transplantations were analyzed by flow cytometry. Colors indicate the treatment in the first transplant. **B** Peripheral blood, bone marrow, and spleen were analyzed by flow cytometry for the B lymphoblastic population, and vehicle-treated mice were divided into the treatment groups of the first transplant and illustrated in a box-and-whisker plot with highlighted median. **C** *BCR::ABL1* expression, normalized to *Gapdh*, in the spleen of transplanted mice. For (**A**–**C**), Mann–Whitney-*U*-test was performed. **D** Overview of mutations found in the kinase domain and myristoyl binding site (aa 268–540) of BCR::ABL1 in secondary transplantations of SCL tTA-BCR::ABL1 mice. The mutations which were detected were assorted into four groups, depending on the treatment of first and secondary recipients, and again into three subgroups, comprising already described mutations in BCR::ABL1 (violet; partly known to provide TKI resistance), previously described mutations of unknown significance in ABL1 (dark red), and undescribed, novel mutations (black) of unknown significance in the kinase domain and myristoyl binding site of BCR::ABL1. aa amino acid, VEH vehicle.

The analysis of the B220+/CD45.1+ lyBC cell population of first vs. second transplantation is summarized in Table S3. Consistent with the primary transplants, asciminib potently suppressed the lyBC population (Fig. 2A) and reduced B220+/CD45.1+ levels in isogenic mice, which had received spleen cells from identical donor mice (Fig. S5A, B). Importantly, the majority of recipients which had received spleen cells from formerly asciminib-treated donors did not develop the lyBC phenotype, demonstrating eradication of malignant LT-HSCs (Fig. 2B). This is also illustrated by decreased *BCR::ABL1* expression (Figs. 2C and S5C). In line with the primary recipients, the percentage of CD11b+/Gr-1+ granulocytes was increased in asciminib-treated mice (Fig. S5D, E). Analysis of the stem cell compartment in BM and spleen revealed no consistent alterations in asciminib- vs. vehicle-treated mice following secondary transplantation (Fig. S6A, B). This finding was expected, given the absence of a rigid hierarchy in LSCs of lyBC, implying that both less mature and more developed blasts may harbor disease-initiating capabilities [14]. However, when dividing the vehicle group of the secondary transplantation into two groups from the first transplantation (vehicle vs. asciminib) and analyzing the LT-HSC compartment in the spleen of these mice, two mice (no. 86 and 83) showed high and three mice showed low percentages of LT-HSCs in the asciminib cohort (Fig. S6C). Mice no. 83 and 86 correspond to the last two mice in Table S3, showing increased percentages of lymphoid blasts. This may mean that increased levels of LT-HSCs in the secondary transplant recipients correspond to a more severe phenotype and less deep response in the respective first transplant recipients.

### Asciminib treatment does not lead to increased mutational load on the *BCR::ABL1* myristoyl binding site

The emergence of drug-resistant mutations in *BCR::ABL1* is a significant risk factor in TKI treatment, and mutations in the myristoyl binding site following asciminib treatment were described [15, 16].

To identify mutations in the KD, including the myristoyl binding site (aa 268–540), BCR::ABL1 cDNA of the secondary transplanted mice was analyzed. The analysis did not cover the N lobe of the KD (aa 242–267). Interestingly, we observed several mutations known to promote TKI resistance (e.g., T315I, L298P, or H396Y), but also undescribed mutations of unknown significance, especially in the region of the myristoyl binding site (Fig. 2D). However, no distinct mutation pattern was noted in both vehicle- and asciminib-treated mice, indicating no direct association with treatment, which is in line with former studies describing genomic instability and KD mutations in CML, and Ph+ ALL patients irrespective of TKI treatment [4, 6, 7, 17, 18]. Our lymphoid Ph+ blast crisis-bearing mice exhibited a relatively high frequency of mutations in the *ABL* gene. This could be due to the fact that this is an inbred mouse strain, and the presence of multiple copies of *BCR::ABL1* in the genome of the SCLtTA-BCR::ABL1 mice may result in the accumulation of numerous mutations in each individual. In addition, reactive oxygen species, known to be elevated in the present mouse model, are potential factors driving genomic instability, leading to TKI-resistant BCR::ABL1 [19, 20].

In conclusion, secondary transplantation revealed a strong response to asciminib with notable efficacy against malignant repopulating stem cells. Only a minority of mice that had been previously treated with asciminib developed lyBC after secondary transplantation and none of the mice that received asciminib treatment after secondary transplantation showed any lyBC phenotype. This argues for continuous dependence of the malignant cells on BCR::ABL1 and against major BCR::ABL1-independent resistance mechanisms, such as efflux receptor activity, upregulated signaling pathways, and alterations in the BM environment. In addition, no selection of KD-mutant clones during asciminib treatment was observed. However, the reason for the heterogeneous response of the secondary recipient mice, who had responded to asciminib after the first transplantation, remains unclear. Together, our preclinical data strongly support further evaluation of asciminib treatment in Ph+ lyBC and ALL, alone or in combination therapy.

### Supplementary information

Supplemental Material_Chatain et al_clean

## Data Availability

The contributions presented in the study are included in the article and the supplemental data and methods. The original datasets used and/or analyzed during the current study are available from the corresponding author on request.
