# Supplementary material for: Asciminib antagonizes transplantable BCR::ABL1-positive lymphoid blast crisis in vivo by targeting malignant stem cells
Source: Leukemia. 2024 Jun 21;38(8):1825–30. doi: 10.1038/s41375-024-02320-9 (PMC11286509; doi:10.1038/s41375-024-02320-9)
Supplement: Supplementary file 1 — Supplemental Material_Chatain et al_clean [file 41375_2024_2320_MOESM1_ESM.docx]

**Asciminib antagonizes transplantable BCR::ABL1-positive lymphoid blast crisis *in vivo* by targeting malignant stem cells**

Nicolas Chatain^1,2,*^, Julian Baumeister^1,2^, Marcelo A. Szymanski de Toledo^1,2^, Dickson W.L. Wong^2,3^, Siddharth Gupta^1,2^, Kristina Pannen^1,2^, Bärbel Junge^1,2^, Tim H. Brümmendorf^1,2^, Peter Boor^2,3^, Steffen Koschmieder^1,2,*^

^1^Department of Hematology, Oncology, Hemostaseology, and Stem Cell Transplanation, Faculty of Medicine, RWTH Aachen University, Aachen, Germany

^2^Center for Integrated Oncology Aachen Bonn Cologne Düsseldorf (CIO ABCD), Aachen, Germany

^3^Institute of Pathology, RWTH Aachen University Clinic, Aachen, Germany

* Correspondence: nchatain@ukaachen.de and skoschmieder@ukaachen.de

COI: S.K. received research funding from Novartis for this preclinical study, received consulting fees, honoraria, and travel/accommodation support from Novartis, and reports participation on advisory boards for Novartis. T.H.B. served as consultant/speaker for Synlab, Incyte, Merck, Novartis, Pfizer, Roche and received research support from Novartis and Pfizer.

**Supplementary Methods**

Table S1. Antibodies

| Rat anti-mouse CD3 Monoclonal Antibody, PE-Cy5 Conjugated (Clone 17A2) | BioLegend | Cat# 100273 |
| --- | --- | --- |
| Rat anti-mouse CD3 Monoclonal Antibody, PE-Cy7 Conjugated (Clone 17A2) | BioLegend | Cat# 100220 |
| Rat anti-CD4 Monoclonal Antibody, PE-Cy5  Conjugated (Clone RM4-5) | BD Biosciences | Cat# 553050 |
| Rat anti-mouse CD8a Monoclonal Antibody, PE/Cy5  Conjugated (Clone 53-6.7) | eBioscience (Invitrogen) | Cat# 100710 |
| Rat anti-mouse/human CD11b Monoclonal Antibody,  PE/Cy5 Conjugated (Clone M1/70) | BioLegend | Cat# 101210 |
| Rat anti-mouse/human CD11b Monoclonal Antibody,  PE/Cy7 Conjugated (Clone M1/70) | BioLegend | Cat# 101216 |
| anti-mouse CD45.1 Monoclonal Antibody,  Phycoerythrin Conjugated (Clone A20) | BioLegend | Cat# 110708 |
| anti-mouse CD45.2 Monoclonal Antibody, PB Conjugated (Clone 104) | BioLegend | Cat# 109820 |
| Rat anti-mouse/human CD45R/B220 Monoclonal  Antibody, PE/Cy5 Conjugated (RA3-6B2) | BioLegend | Cat# 103210 |
| Rat anti-mouse/human CD45R/B220 Monoclonal  Antibody, PB Conjugated (RA3-6B2) | BioLegend | Cat# 103230 |
| Hamster anti-mouse CD48 Monoclonal Antibody, FITC Conjugated (HM48-1) | Biolegend | Cat# 103404 |
| Rat anti-mouse CD117/c-kit Monoclonal Antibody,  APC / Cy7 Conjugated (Clone 2B8) | Biolegend | Cat# 105826 |
| Rat anti-mouse CD150 (SLAM) Monoclonal Antibody,  APC Conjugated (Clone TC15-12F12.2) | Biolegend | Cat# 115910 |
| Rat anti-mouse Ly-6G/Ly-6C (Gr-1) Monoclonal  Antibody, PE/Cy5 Conjugated (RB6-8C5) | BioLegend | Cat# 108410 |
| Rat anti-mouse Ly-6G/Ly-6C (Gr-1) Monoclonal  Antibody, FITC Conjugated (Clone RB6-8C5) | BioLegend | Cat# 108405 |
| Rat anti-mouse Sca-1 Monoclonal  Antibody, PE/Cy7 Conjugated (Clone D7) | Biolegend | Cat# 108113 |
| Rat anti-mouse TER-119/Erythroid Cells Monoclonal  Antibody, PE/Cy5 Conjugated (Clone TER-119) | BioLegend | Cat# 116210 |

Table S2. Primer

| For endpoint PCR | |
| --- | --- |
| B/A KD fwd | GCCTGGCCTACAACAAGTTCTC |
| B/A KD rev | GTGTAGGTGTCCCCTGTCATC |
| For RT-qPCR | |
| *BCR::ABL1* for | TCGTCCACTCAGCCACTGG |
| *BCR::ABL1* rev | GGCTTCACTCAGACCCTGA |
| *Gapdh* fwd | TTGTGCAGTGCCAGCCTC |
| *Gapdh* rev | CCAATACGGCCAAATCCG |

Table S3. Aberrant B cell population in primary and secondary transplantation, stratified by treatment and analyzed organ.


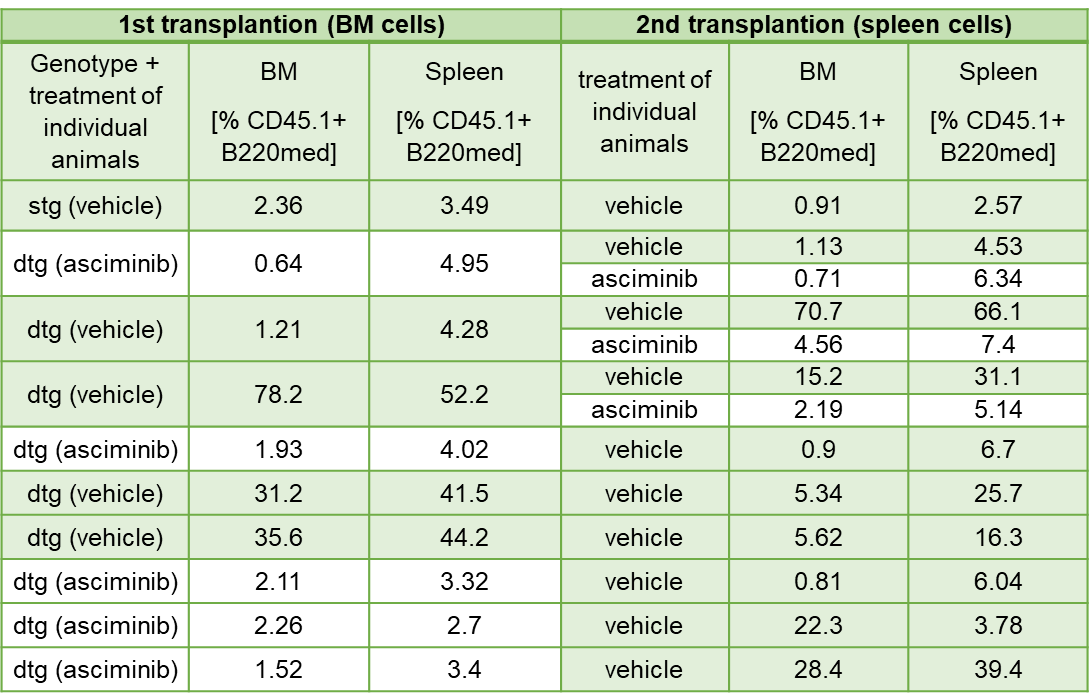
Asciminib treatment in white; vehicle treatment in green; B220med – medium high B220 population; BM – bone marrow

**Mouse experiments**

Bone marrow (BM) cells were harvested from 5-FU-treated double transgenic (dtg) or single transgenic (stg; controls) donor mice (CD45.1+) and transplanted into lethally irradiated recipient mice (CD45.2+). Tetracycline (tet) was removed from the drinking water to induce BCR::ABL1 expression for two weeks, and mice were treated daily for 5 weeks with ABL001 or vehicle control by oral gavage (30 mg/kg body weight) (Figure S1A). Secondary transplantations were performed with frozen splenocytes of the first transplantations and the mice were subjected to further ABL001 treatment (daily, 5 weeks) (Figure S1B).

For final analysis, femurs and tibias were flushed with PBS containing 2% fetal calf serum (FCS) and the BM cells were passed through a 70 µm filter and counted (BM refers to the cells flushed from 2 tibias and 2 femurs/mouse). Spleen tissue was minced and strained through a 70 µm cell strainer to obtain single cell suspensions. After lysis of erythrocytes with 2 successive incubations (2 min, RT) in ACK-lysis buffer (0.15 M NH4Cl, 1 mM KHCO3, 0.1 mM Na2EDTA, pH 7,3), cells were washed with PBS/2% FCS, counted and subjected to antibody staining for flow cytometry.

**Immunohistochemical analysis**

The bone and spleen were isolated from mice and fixed in 4% formalin for 24 hours, followed by tissue processing steps with dehydration and paraffin embedment.. Tissue sections (1 µm), obtained from formalin-fixed, paraffin-embedded (FFPE) blocks, were first deparaffinized and rehydrated, followed by heat-induced antigen retrieval with citrate buffer (Vector Laboratories; H-3300) then 3% H2O2 and rat serum (Thermo Fisher Scientific; 31888) incubation. The anti-B220 primary antibody (Biolegend; 103227) and, subsequently, the biotinylated anti-rat secondary antibody (Vector Laboratories; BA-4001-.5) were applied to the tissues. The staining signal was then amplified with ABC-HRP Kit (Vector Laboratories; PK-6100) and developed with ImmPACT® VIP Substrate Kit (Vector Laboratories; SK-4605).

**Supplementary Figures**


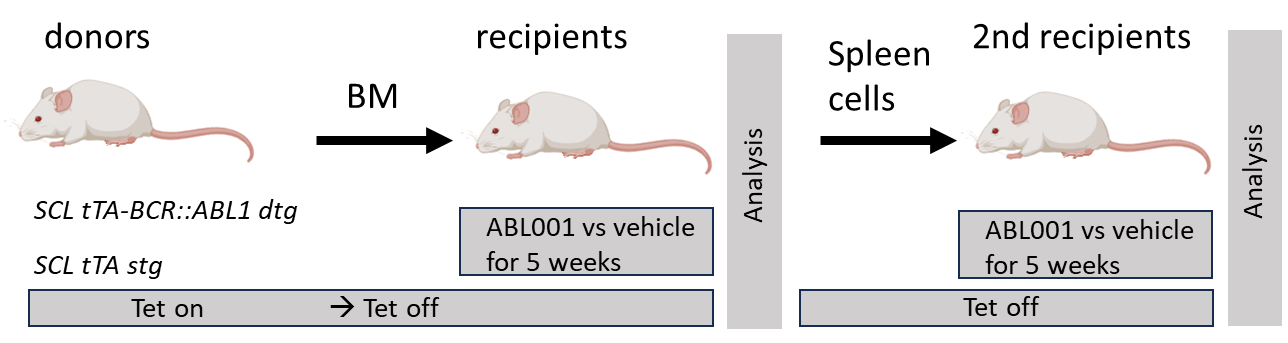


**A**

**B**

**Figure S1. Schematic overview of experimental design. (A)** 5-FU-treated bone marrow (BM) cells were harvested from double transgenic (dtg) or single transgenic (stg) donor mice (CD45.1+) and transplanted into lethally irradiated recipient mice (CD45.2+; 1x10^6^ per mouse). Tetracycline (tet) was removed from the drinking water one week after transplantation to induce *BCR::ABL1* expression for one week, and, subsequently, mice were treated daily with ABL001 or vehicle control by oral gavage (30 mg/kg body weight) for 5 weeks (1 week tet on + 1 week tet off + 5 weeks of treatment). (B) Secondary transplantations were performed in the absence of tet, using splenocytes (1x10^6^ per mouse) were performed to analyze whether ABL001 treatment suppressed BCR::ABL1-positive repopulating malignant stem cells. In some cases, mouse pairs received the cells of the same donors. Additionally, we assessed potential ABL001-resistant subclones after secondary transplantation by subjecting the mice to further ABL001 treatment 2 weeks after transplantation for 5 weeks.


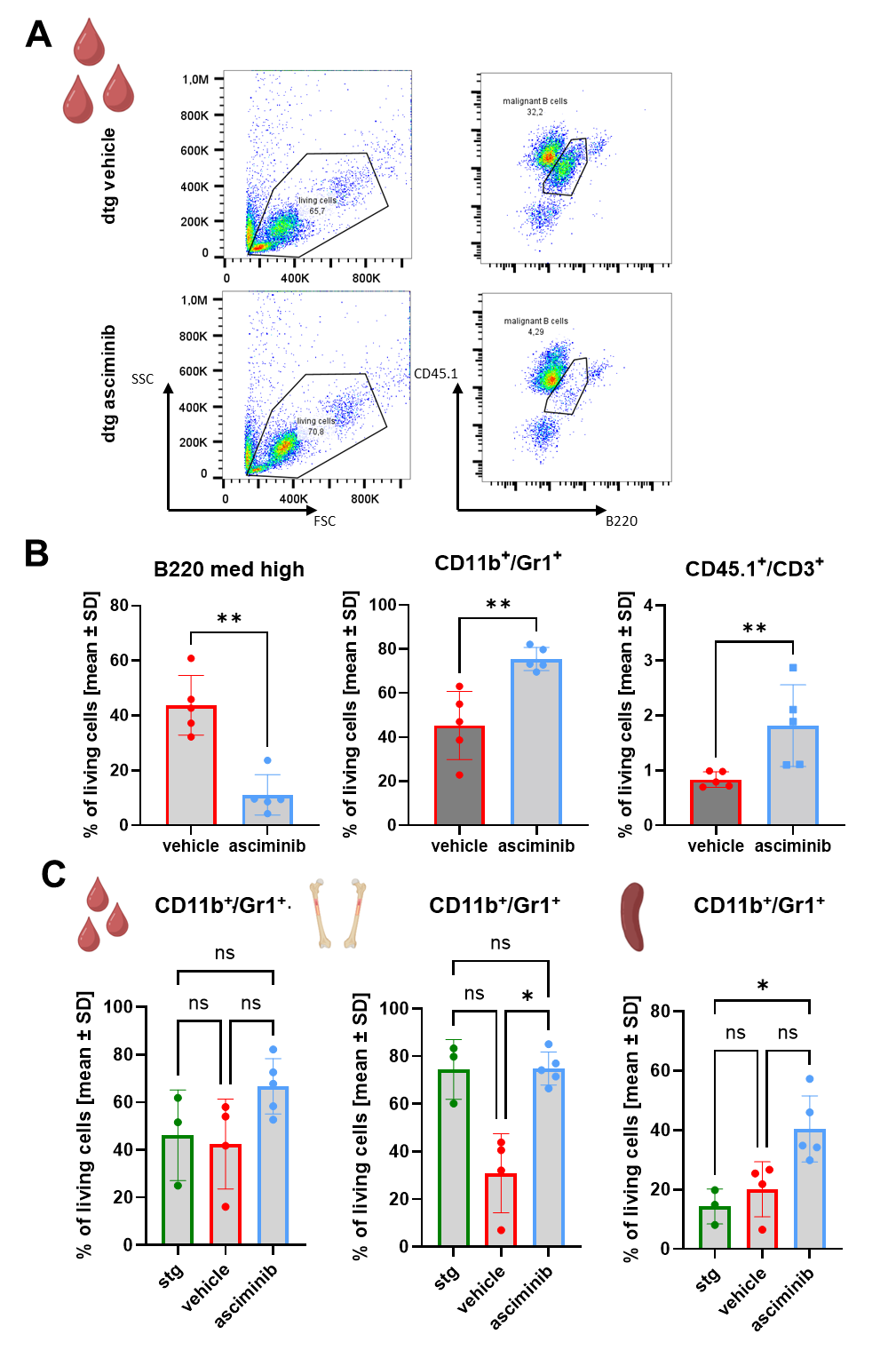


**Figure S2. Two weeks after induction of *BCR::ABL1* expression by tetracycline withdrawal, and one week after start of ABL001 treatment, peripheral blood samples were analyzed by flow cytometry. (A)** Flow cytometry dot plots are presented to illustrate the development of the CD45.1+/B220+ intermediate cell population, representing the BCR::ABL1-positive lymphoid blast crisis population. dtg – double transgenic **(B)** Bar plots and statistical analysis (Mann-Whitney-U-test) of the percentage of CD45.1/B220 medium high (B220 med high), CD11b+/Gr1+ (granulocytes) and CD3 (T cells) in peripheral blood (PB). **p < 0.01; SD – standard deviation. **(C)** Relative CD11b+/Gr1+ granulocyte population in PB, bone marrow and spleen after final analysis. Kruskal-Wallace and Dunn’s multiple comparisons test was performed. *p < 0.05; SD – standard deviation, stg – single transgenic control mice.


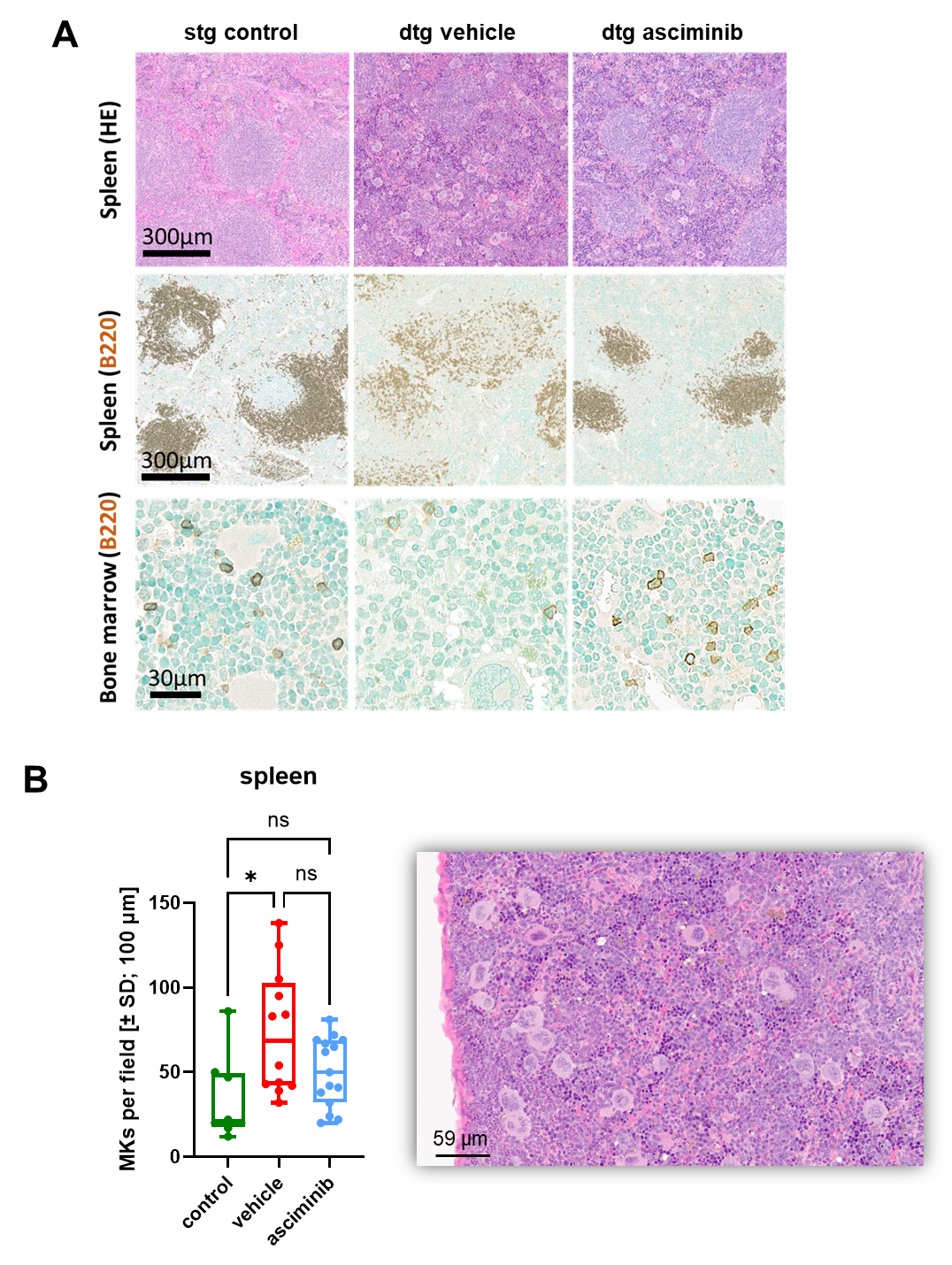


**Figure S3. Normalization of bone marrow and spleen histology as well as megakaryocyte infiltration upon ABL001 treatment. (A)** Exemplary HE and B220 staining of spleen and B220 staining of bone marrow for primary transplanted mice. stg – single transgenic control mice; dtg – double transgenic mice. (B) left: Megakaryocytes (MKs) were counted in three 100 µm fields per mouse of HE stained spleen histologies. Box-and-whisker plot is shown with marked median. Kruskal-Wallace and Dunn’s multiple comparisons test was performed. *p < 0.05; Right: Illustration of massive MK infiltration of the spleen. Exemplary HE staining of a vehicle treated mouse. ns – not significant; SD – standard deviation


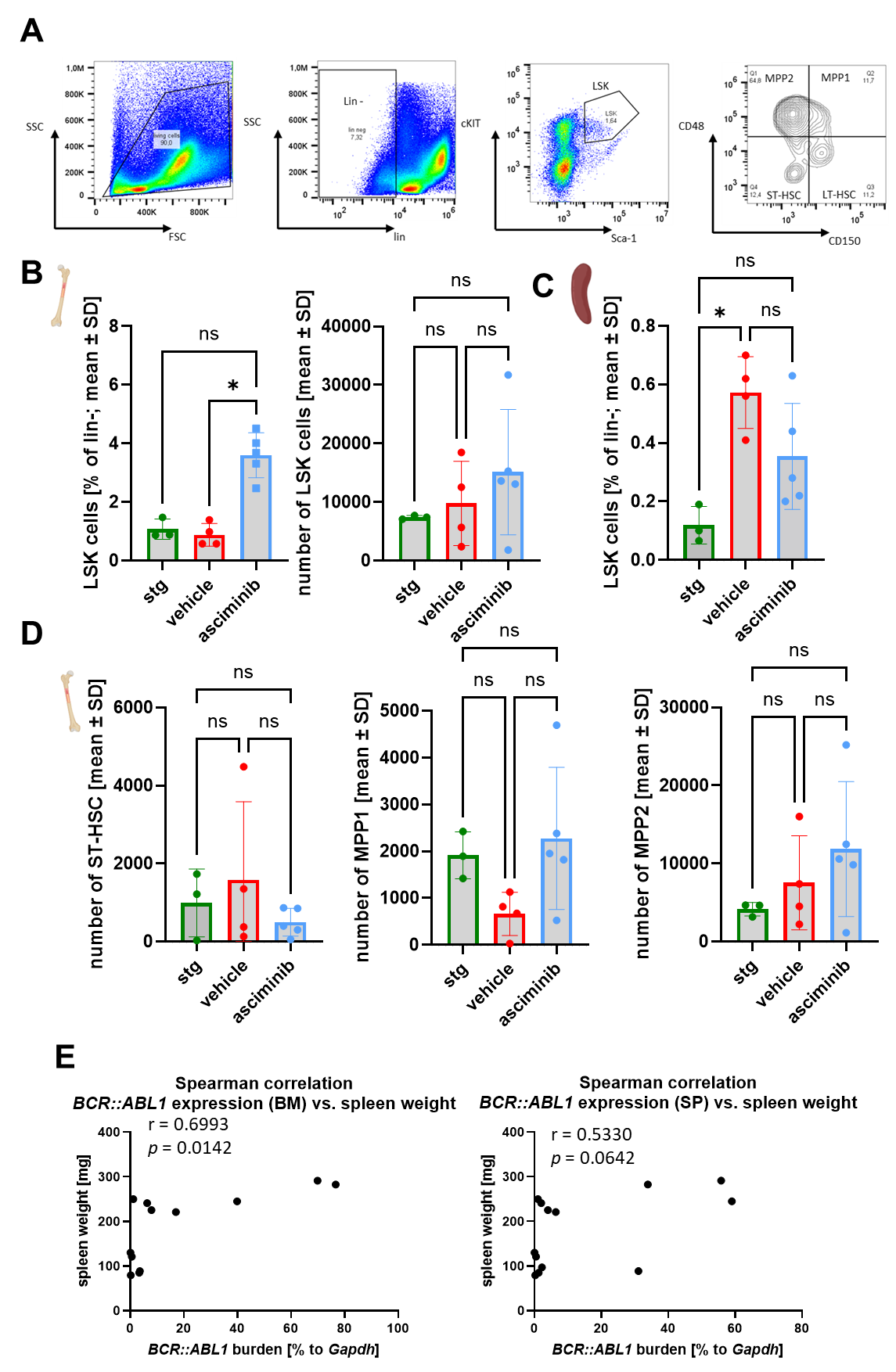


**Figure S4. Analysis of the stem cell compartment in bone marrow and spleen of primary transplanted mice. (A)** Gating strategy for the analysis of the lin- Sca-1+ cKIT+ (LSK) population and long-term as well as short-term hematopoietic stem cells (LT-HSC, ST-HSC) and multipotent progenitor populations MPP1 and MPP2. **(B)** Analysis of the lin- Sca1+ cKIT+ (LSK) cell population in BM (% of lin- cells and total numbers) and **(C)** SP (% of lin- cells). **(D)** In the LSK population, total number of ST-HSC, MPP1 and MPP2 cells was analyzed. For **B**, **C** and **D** One-way ANOVA test (Kruskal-Wallace and Dunn’s multiple comparisons test) was used for statistical analysis. *p < 0.05, ns = not significant. **(E)** Spearman correlations of BCR::ABL1 expression and spleen weight in bone marrow (BM) and spleen (SP).


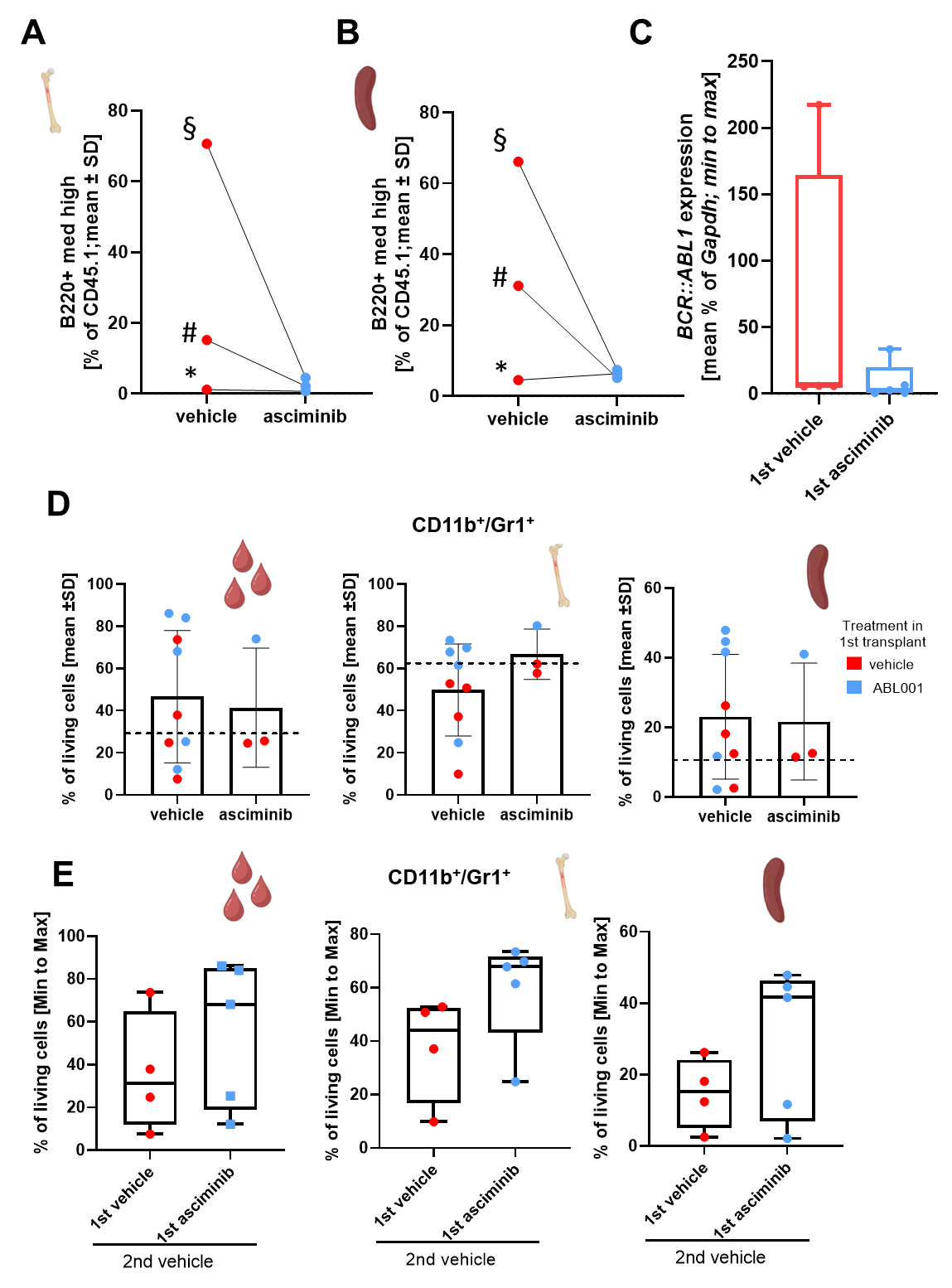
**Figure S5. Analysis of secondary transplantation showing ABL001 efficacy. (A) (B)** Analysis of the B220 medium high/CD45.1+ population of the syngenic transplanted mouse pairs with the same splenocytes of the first transplantation in bone marrow and spleen. * was in the ABL001 group, # and § were in the vehicle group of the first transplantation.  **(C)** Box-and-whisker plot with highlighted median of *BCR::ABL1* expression in the spleen of secondary transplantation only analyzing the vehicle group divided into the treatment groups of the first transplantation. **(D)**, **(E)** CD11b+/Gr1+ granulocytes of the second transplantation in peripheral blood, bone marrow and spleen all in one graph **(D)** with colors marking the differential treatment groups of the primary transplantation (scatter dot plot with SD and dotted line to highlight control mouse) or **(E)** comparison of the treatment groups of the first transplantation in the vehicle group of the secondary transplantation (Box-and-whiskers plots with Min, Max and marked median).


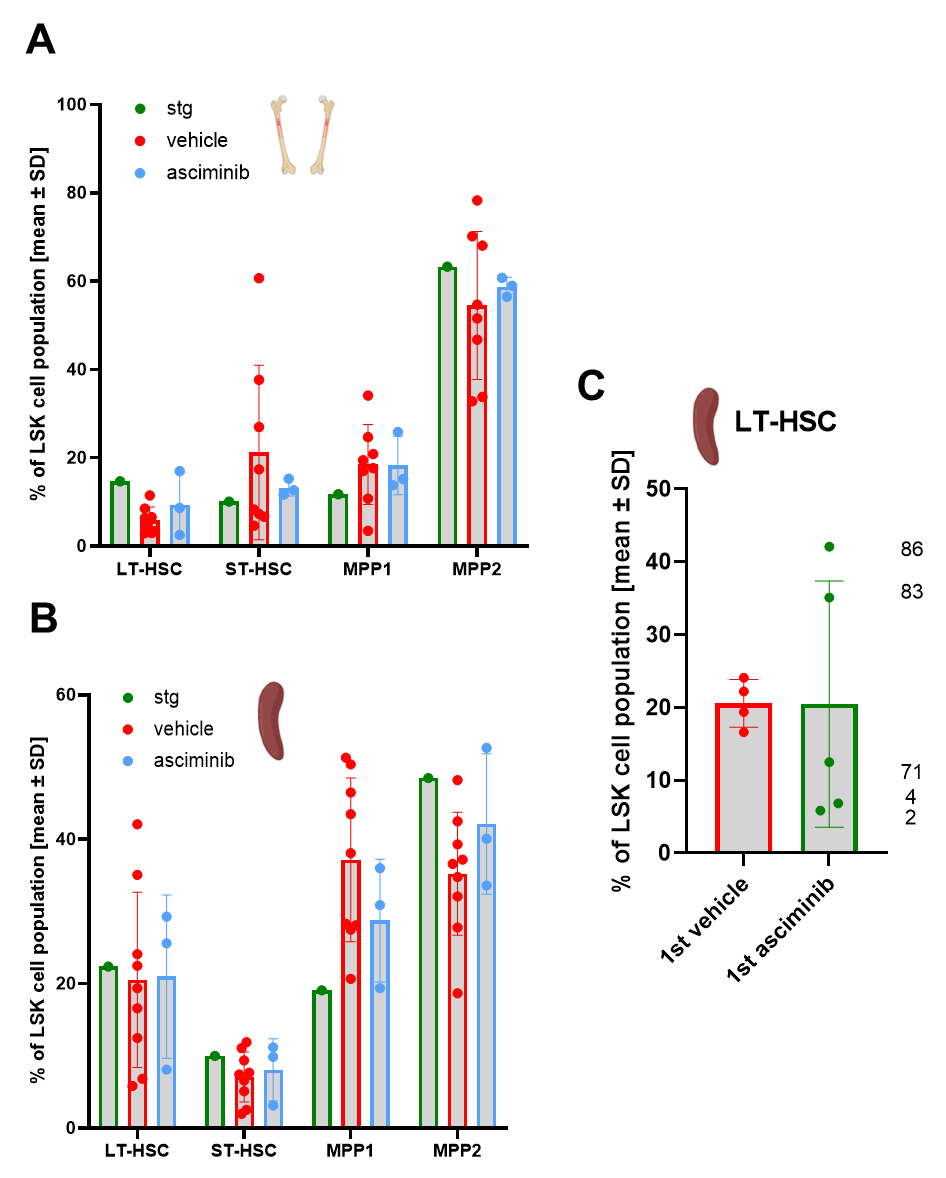


**Figure S6. Varying changes in the stem cell compartment of secondary transplantation.** In the lin- Sca1+ cKIT+ (LSK) cell population, the percentage of the LT-HSCs, ST-HSCs, MPP1 and MPP2 cells was analyzed in **(A)** bone marrow and **(B)** spleen. One-way ANOVA test (Kruskal-Wallace) was used for statistical analysis. **(C)** Vehicle-treated recipients of the second transplantation were divided into the treatment groups of the first transplantation (1st vehicle vs. 1st asciminib), and LT-HSCs in the spleen were analyzed (% of LSKs). Individual mouse numbers of the asciminib treatment group are provided next to the green bar for reference to the main text.
